# Interpretable prediction of coronary heart disease risk in adults over 50 with accelerated aging using 45 dietary nutrients

**DOI:** 10.3389/fnut.2025.1666644

**Published:** 2025-09-18

**Authors:** Zhi-qiang Yang, Xiao-hong Zhang

**Affiliations:** Department of Cardiology, The Third Affiliated Hospital of Anhui Medical University, Hefei, China

**Keywords:** dietary nutrients, aging, phenotypic age acceleration, coronary heart disease, NHANES

## Abstract

**Background:**

The relationship between dietary nutrient intake and coronary heart disease (CHD) risk among older adults with accelerated aging remains inadequately understood.

**Methods:**

This study analyzed data from seven cycles of the National Health and Nutrition Examination Survey (NHANES) conducted in the United States between 2005 and 2018. Weighted Quantile Sum (WQS) regression was employed to evaluate the association between dietary nutrient mixtures and CHD risk in individuals aged 50 and older with accelerated aging. Additionally, six machine learning models were developed, with SHAP and LIME algorithms applied to assess the contribution of individual nutrients to CHD risk.

**Results:**

In the fully adjusted model, dietary nutrient mixtures were inversely associated with CHD risk in older adults experiencing accelerated aging (adjusted OR = 0.90, 95% CI: 0.81–0.99, *p* = 0.048). Both SHAP and LIME analyses consistently identified vitamin B12 and lutein + zeaxanthin as protective nutrients, independent of demographic adjustments.

**Conclusion:**

Among adults aged 50 and older with accelerated aging, higher intake of specific dietary nutrients was associated with reduced CHD risk. Of the machine learning models tested, the random forest algorithm demonstrated the strongest predictive performance. SHAP and LIME analyses jointly highlighted vitamin B12 and lutein + zeaxanthin as key contributors to the reduced CHD risk in this high-risk population.

## Introduction

Coronary heart disease (CHD) remains a leading cause of morbidity and mortality in older adults, particularly those experiencing accelerated aging—a condition in which biological age exceeds chronological age. This discrepancy indicates increased vulnerability to age-related diseases, including CHD (1). Accelerated aging is often assessed using phenotypic age acceleration (PhenoAgeAccel), a biomarker-based metric that reflects physiological decline driven by systemic inflammation and oxidative stress, both of which are central to CHD pathogenesis (2, 3). Given that diet is a modifiable risk factor, understanding its role in CHD among individuals with accelerated aging has substantial clinical and public health significance.

Accelerated aging is characterized by a faster rate of biological deterioration, typically quantified by phenotypic age. This biomarker integrates chronological age with nine clinical indicators—such as glucose, C-reactive protein, and creatinine—to capture metabolic, inflammatory, and organ function status (4). PhenoAgeAccel is defined as the difference between phenotypic and chronological age; positive values denote accelerated aging. This metric is a strong predictor of morbidity and mortality, underscoring its relevance in aging research and clinical risk assessment (1, 4).

The prevalence of accelerated aging is particularly high among adults aged 50 and older and is influenced by lifestyle behaviors, socioeconomic status, comorbidities, and environmental exposures (5). Favorable cardiovascular health is typically associated with negative PhenoAgeAccel scores, whereas poor cardiovascular profiles are linked to positive values (6). Modifiable factors—such as smoking, physical inactivity, poor diet, and obesity—contribute significantly to accelerated aging and increase the risk of conditions like diabetes, frailty, cognitive decline, and cardiovascular disease (2, 7). Additionally, genetic predisposition and exposure to pollutants may further exacerbate biological aging (8).

Mechanistically, accelerated aging increases CHD risk through heightened inflammation and oxidative stress, which promote endothelial dysfunction and atherogenesis (3, 9). In a study of 609 patients with multivessel coronary artery disease, higher phenotypic age was significantly associated with increased all-cause mortality, reinforcing its prognostic relevance in cardiovascular care (10). Similarly, epigenetic age acceleration has been linked to unfavorable cardiometabolic profiles and higher cardiovascular risk scores, especially in high-risk populations (11). These findings emphasize the critical role of biological aging in CHD development.

Dietary nutrients play a key role in regulating systemic inflammation and oxidative stress—both integral to aging and CHD pathogenesis (12, 13). Several nutrients, such as vitamin E, vitamin C, and omega-3 fatty acids, have been widely studied for their antioxidant and anti-inflammatory effects (14–18). Vitamin E, a lipid-soluble antioxidant, protects cell membranes by neutralizing reactive oxygen species (ROS) and suppressing pro-inflammatory cytokines like interleukin-6 (IL-6) and tumor necrosis factor-alpha (TNF-α) (14, 15). High doses (≥700 mg/day) have been associated with reductions in C-reactive protein (CRP) levels and improvements in insulin resistance (14), with well-documented protective effects against oxidative stress–related diseases, including CHD (15). Vitamin C, a water-soluble antioxidant, scavenges ROS, regenerates oxidized vitamin E, and lowers inflammatory markers such as CRP and IL-6 (16, 17). These actions help reduce oxidative damage in conditions like metabolic syndrome and may lower CHD risk (17). Omega-3 fatty acids—especially eicosapentaenoic acid (EPA) and docosahexaenoic acid (DHA)—reduce inflammation by inhibiting pro-inflammatory eicosanoids and cytokines while promoting the synthesis of pro-resolving mediators (18, 19). Though these nutrients have been associated with reduced cardiovascular mortality and nonfatal myocardial infarction, data specific to older adults remain limited (20).

These bioactive compounds also modulate key inflammatory pathways, such as NF-κB and the NLRP3 inflammasome, and intersect with diseases influenced by chronic inflammation and oxidative stress—including cardiovascular disease, diabetes, and neurodegenerative disorders (14–19). However, their efficacy may vary depending on factors such as dosage, bioavailability, and individual metabolic status.

Although nutrients like vitamin E, vitamin C, and omega-3 fatty acids show potential for mitigating inflammation and oxidative stress in the general population, their specific effects on CHD risk in older adults with accelerated aging remain unclear (12, 13). Potential mechanisms include reducing endothelial dysfunction and slowing atherogenesis through antioxidant and anti-inflammatory actions (9, 13). Nevertheless, few clinical trials have focused on this high-risk subgroup, and the complex interplay between biological aging, nutrient metabolism, and cardiovascular outcomes remains underexplored (21).

In response to this knowledge gap, the present study analyzed NHANES data to examine the association between various dietary nutrients—including carbohydrates, fiber, vitamins, and minerals—and CHD risk in individuals aged 50 and above with elevated PhenoAgeAccel. By integrating machine learning models capable of capturing nonlinear and interactive nutrient effects (22, 23), the study aimed to identify key dietary predictors and inform targeted nutritional strategies for CHD prevention in this aging population.

## Materials and methods

### Study population

**Figure 1:**
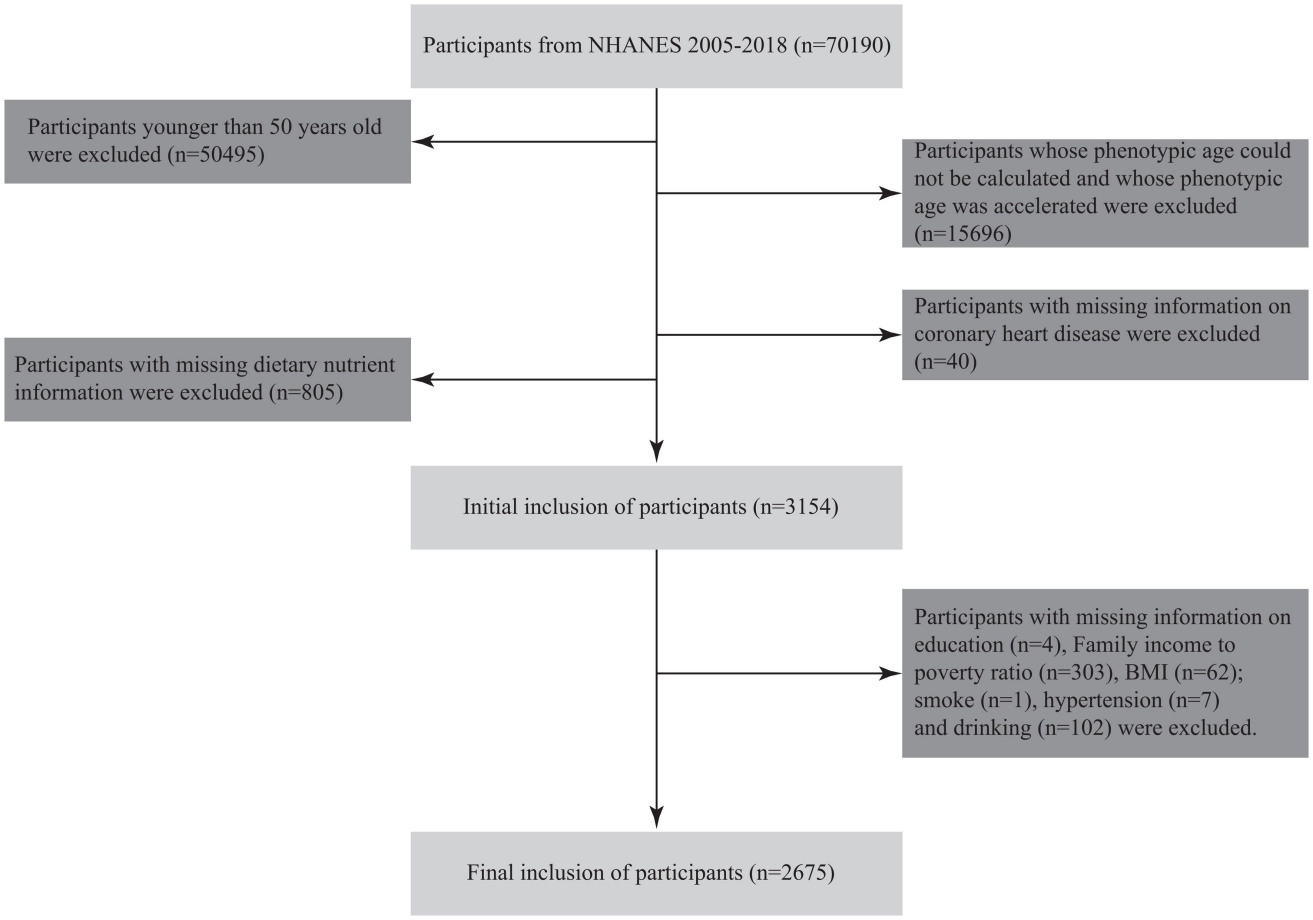
Flowchart illustrating the study design and participant selection process.

The NHANES, conducted by the National Center for Health Statistics (NCHS), evaluates the health and nutritional status of the non-institutionalized U. S. population. This study utilized NHANES data from 2005 to 2018, initially comprising 70,190 participants. After applying exclusion criteria, 67,515 individuals were removed for the following reasons: (1) age under 50 years (n = 50,495); (2) missing data needed for calculating phenotypic age and PhenoAgeAccel (n = 15,696); (3) absence of CHD information (n = 40); (4) missing dietary micronutrient data (n = 805); (5) missing education information (n = 4); and (6) missing data on PIR, BMI, smoking, hypertension, or alcohol use (n = 475). The final analytical sample included 2,675 participants (Figure 1).

### Assessment of dietary micronutrients

Micronutrient intake, including vitamins and carbohydrates, was assessed using 24-h dietary recall data collected during the first and second interview days of NHANES. The first interview was conducted in person at the Mobile Examination Center (MEC), while the second was administered by telephone several days later. Both interviews were conducted by trained professionals using the Automated Multiple-Pass Method (AMPM), which enables comprehensive and standardized documentation of food and beverage consumption.

### Assessment of coronary heart disease

CHD status was determined based on participants’ self-reported physician diagnoses, including “coronary heart disease,” “angina pectoris,” and “heart attack.” Data were collected through in-person interviews conducted by trained personnel using the Computer-Assisted Personal Interviewing (CAPI) system. Relevant items were derived from the Medical Conditions Questionnaire (MCQ). Participants were classified as having CHD if they responded “yes” to any of the diagnoses of “coronary heart disease,” “angina pectoris,” or “heart attack.”

### Assessment of phenotypic age and PhenoAgeAccel

Phenotypic age was calculated using the algorithm developed by Levine et al., which integrates chronological age with nine biomarkers: albumin, creatinine, glucose, log-transformed C-reactive protein (CRP), lymphocyte percentage, mean cell volume, red cell distribution width, alkaline phosphatase, and white blood cell count. The calculation was performed using a Cox proportional hazards elastic net model with 10-fold cross-validation. Phenotypic age acceleration was defined as the residual from a linear regression of phenotypic age on chronological age. Negative values indicated a biologically younger state, while positive values indicated a biologically older state. The complete formula used in the calculation is provided below:


$$\text{Phenotypic}\;\mathrm{Age}=141.50+\frac{\ln[-0.00553\times \ln(1-M)]}{0.09165}$$

Where


$$M=1-\exp(\frac{-1.51714\times \exp(\mathrm{xb})}{0.0076927})$$

$$\begin{array}{l} \mathrm{xb}=-19.907+0.0804\times \text{ChronologicalAge}-0.0336\\ \times \text{Albumin}+0.0095\times \text{Creatinine}+0.1953\\ \times \text{Glucose}+0.0954\times \ln(\mathrm{CRP})-0.0120\\ \times \text{LymphocytePercent}+0.0268\times \text{MeanCellVolume}\\ +0.3306\times \text{RedCellDistributionWidth}+0.00188\\ \times \text{AlkalinePhosphatase}+0.0554\times \text{WhiteBloodCellCount} \end{array}$$

### Covariates

Sociodemographic and lifestyle covariates included age, sex, race/ethnicity (Mexican American, Other Hispanic, Non-Hispanic White, Non-Hispanic Black, Other Race), education level (<9th grade, 9–11th grade, high school diploma/GED, some college/AA degree, ≥college graduate), family income-to-poverty ratio (PIR), BMI, smoking status, alcohol use, hypertension, and diabetes. Hypertension was defined as a self-reported physician diagnosis and current use of antihypertensive medication. Diabetes was defined as physician-diagnosed diabetes, a 2-h OGTT glucose ≥11.1 mmol/L, or fasting glucose ≥7.0 mmol/L. Prediabetes was defined as a prior diagnosis or intermediate glucose levels (2-h glucose 7.8–11.1 mmol/L or fasting glucose 6.1–6.9 mmol/L). Smoking status was categorized as never/long-term former (never smoked or quit >1 year ago) or current smoker (smoked within the past 30 days, smoked upon waking, or smoked >2 cigarettes/day after quitting). Drinking status was classified as lifetime abstainer (<12 drinks in lifetime) or current drinker (≥12 drinks/year or drinking on >6 occasions in the past 12 months). BMI was calculated as weight in kilograms divided by height in meters squared (kg/m^2^).

### Feature preprocessing and selection for machine learning

A total of 56 features were considered, including 49 continuous and 7 categorical variables. Features with variance inflation factors (VIF) > 3, adjusted for degrees of freedom, were excluded to reduce multicollinearity. To address class imbalance and improve recognition of the minority class, the Synthetic Minority Over-sampling Technique (SMOTE) was applied. SMOTE generates synthetic data points by interpolating between existing samples and their k-nearest neighbors, thereby enhancing dataset balance (Supplementary Figure 1). All variables were standardized to minimize disproportionate influence due to differing scales.

Feature selection was performed using the Boruta algorithm, a random forest-based method that evaluates feature importance over 500 iterations by comparing real features with randomized shadow features. Only features classified as “confirmed” were retained for model development.

### Statistical analyses

All statistical procedures followed NHANES analytical guidelines. Continuous variables were presented as means ± standard deviations (SD), and categorical variables as frequencies and percentages. Group comparisons used chi-square tests for categorical variables and Student’s t-tests for continuous variables. To examine the joint effect of dietary micronutrient mixtures on CHD in older adults with accelerated aging, Weighted Quantile Sum (WQS) regression was employed. Weights for each nutrient component were estimated using 1,000 bootstrap iterations. Data were randomly split into training (60%) and testing (40%) sets to enhance model reliability.

To avoid overfitting, a 6:4 training-validation split was maintained throughout model construction. Six machine learning models were developed using the MLR3 framework: Random Forest, LightGBM, K-Nearest Neighbors (KNN), Naive Bayes, Support Vector Machine (SVM), and XGBoost. Random Forest: Aggregates multiple decision trees to deliver robust predictions and is inherently resistant to overfitting. LightGBM: An efficient gradient-boosted decision tree model optimized for speed, memory usage, and parallel computation. K-NN: Classifies samples based on proximity to neighbors; performs well on small or non-linear datasets. Naive Bayes: A fast, probabilistic classifier based on Bayes’ theorem, effective even with missing values. SVM: Identifies optimal hyperplanes to separate classes, particularly effective in high-dimensional data. XGBoost: A highly efficient gradient boosting framework that balances accuracy and computational efficiency.

Model performance was evaluated using standardized datasets and the following six metrics: accuracy, F beta score, area under the ROC curve (AUC-ROC), sensitivity, specificity, and area under the precision-recall curve (AUC-PR). AUC-ROC was used as the primary evaluation metric. Ten-fold cross-validation was applied to enhance generalizability. ANOVA and Kruskal-Wallis H tests were used to compare model performance metrics.

To enhance model interpretability, SHAP (SHapley Additive exPlanations) and LIME (Local Interpretable Model-Agnostic Explanations) were used. SHAP, grounded in cooperative game theory, quantifies each feature’s contribution by considering all feature combinations, thus offering transparent and consistent interpretations. LIME creates interpretable local approximations (e.g., linear models) to explain predictions made by complex models.

All statistical analyses were conducted using IBM SPSS Statistics (version 24.0) and R (version 4.5.0). A two-tailed *p*-value < 0.05 was considered statistically significant.

## Results

### Participant characteristics by coronary heart disease status

Table 1 presents baseline characteristics of older adults aged 50 years and above with accelerated aging, stratified by CHD status. A total of 2,675 participants from NHANES 2005–2018 were included. The mean age was 65.62 years (SD = 9.38), comprising 902 females (33.72%) and 1,773 males (66.28%). Among them, 565 individuals were diagnosed with CHD, with a higher mean age of 69.53 years (SD = 8.52).

**Table 1:** Baseline characteristics of the study participants.

Characteristic	Overall *N* = 2,675	No Coronary heart disease *N* = 2,110	Coronary heart disease *N* = 565	*p*
Age (year)^a^, Mean ± SD	65.62 ± 9.38	64.57 ± 9.33	69.53 ± 8.52	<0.001
Sex^b^, *n* (%)				<0.001
Female	902 (33.72%)	754 (35.73%)	148 (26.19%)	
Male	1,773 (66.28%)	1,356 (64.27%)	417 (73.81%)	
Race/ethnicity^b^, *n* (%)				<0.001
Mexican	354 (13.23%)	303 (14.36%)	51 (9.03%)	
Other Hispanic	254 (9.50%)	207 (9.81%)	47 (8.32%)	
Non-Hispanic White	1,253 (46.84%)	921 (43.65%)	332 (58.76%)	
Non-Hispanic Black	652 (24.37%)	552 (26.16%)	100 (17.70%)	
Other Race	162 (6.06%)	127 (6.02%)	35 (6.19%)	
Education^b^, *n* (%)				0.569
Less Than 9th	378 (14.13%)	289 (13.70%)	89 (15.75%)	
9–11th	418 (15.63%)	328 (15.55%)	90 (15.93%)	
High School	700 (26.17%)	554 (26.26%)	146 (25.84%)	
Some College	741 (27.70%)	583 (27.63%)	158 (27.96%)	
College Graduate	438 (16.37%)	356 (16.87%)	82 (14.51%)	
Family income to poverty ratio^a^, Mean ± SD	2.36 ± 1.53	2.39 ± 1.55	2.24 ± 1.46	0.082
BMI^a^, Mean ± SD	31.62 ± 7.65	31.59 ± 7.74	31.74 ± 7.27	0.277
Smoking status^b^, *n* (%)				0.223
No	2,075 (77.57%)	1,626 (77.06%)	449 (79.47%)	
Yes	600 (22.43%)	484 (22.94%)	116 (20.53%)	
Drinking status^b^, *n* (%)				0.890
No	336 (12.56%)	266 (12.61%)	70 (12.39%)	
Yes	2,339 (87.44%)	1,844 (87.39%)	495 (87.61%)	
Hypertension^b^, *n* (%)				<0.001
No	892 (33.35%)	780 (36.97%)	112 (19.82%)	
Yes	1,783 (66.65%)	1,330 (63.03%)	453 (80.18%)	
Diabetes^b^, *n* (%)				<0.001
No	1,230 (45.98%)	1,025 (48.58%)	205 (36.28%)	
Yes	1,100 (41.12%)	816 (38.67%)	284 (50.27%)	
Borderline	345 (12.90%)	269 (12.75%)	76 (13.45%)	
Energy^a^, Mean ± SD	1,971.27 ± 918.15	2,008.67 ± 934.49	1,831.58 ± 840.57	<0.001
Protein^a^, Mean ± SD	75.36 ± 39.56	76.52 ± 40.27	71.01 ± 36.53	0.002
Carbohydrate^a^, Mean ± SD	235.33 ± 115.75	240.12 ± 117.63	217.45 ± 106.66	<0.001
Total Sugar^a^, Mean ± SD	103.11 ± 71.17	105.44 ± 72.29	94.39 ± 66.16	<0.001
Dietary fiber^a^, Mean ± SD	15.47 ± 9.64	15.71 ± 9.77	14.57 ± 9.06	0.022
Total Fat^a^, Mean ± SD	77.20 ± 44.99	78.21 ± 45.62	73.43 ± 42.40	0.033
Saturated fatty acids^a^, Mean ± SD	25.12 ± 16.12	25.42 ± 16.55	23.97 ± 14.32	0.176
Monounsaturated fatty acids^a^, Mean ± SD	27.60 ± 16.77	27.92 ± 17.01	26.43 ± 15.80	0.090
Polyunsaturated fatty acids^a^, Mean ± SD	17.40 ± 11.91	17.68 ± 11.93	16.38 ± 11.78	0.006
Cholesterol^a^, Mean ± SD	291.70 ± 231.02	292.37 ± 232.67	289.21 ± 224.97	0.659
Vitamin E as alpha-tocopherol^a^, Mean ± SD	7.50 ± 5.63	7.57 ± 5.67	7.24 ± 5.50	0.083
Alpha-tocopherol^a^, Mean ± SD	0.53 ± 2.91	0.48 ± 2.84	0.72 ± 3.17	0.033
Retinol^a^, Mean ± SD	395.43 ± 618.31	394.87 ± 662.97	397.55 ± 411.10	0.123
Vitamin A^a^, Mean ± SD	578.41 ± 707.99	582.42 ± 754.49	563.44 ± 497.50	0.267
Alpha-carotene^a^, Mean ± SD	345.59 ± 898.35	352.58 ± 918.80	319.46 ± 817.71	0.039
Beta-carotene^a^, Mean ± SD	1,982.71 ± 3,694.98	2,033.20 ± 3,816.71	1,794.13 ± 3,195.70	0.445
Beta-cryptoxanthin^a^, Mean ± SD	86.14 ± 264.65	87.98 ± 289.00	79.30 ± 140.33	0.529
Lycopene^a^, Mean ± SD	4,565.04 ± 8,506.70	4,499.93 ± 8,441.73	4,808.19 ± 8,748.41	0.562
Lutein+zeaxanthin^a^, Mean ± SD	1,338.33 ± 3,167.69	1,394.34 ± 3,406.23	1,129.16 ± 2,032.86	0.406
Thiamin(Vitamin B1)^a^, Mean ± SD	1.50 ± 0.84	1.51 ± 0.85	1.48 ± 0.81	0.584
Riboflavin(Vitamin B2)^a^, Mean ± SD	1.94 ± 1.15	1.94 ± 1.18	1.93 ± 1.05	0.476
Niacin^a^, Mean ± SD	23.22 ± 14.03	23.56 ± 14.41	21.94 ± 12.45	0.022
Vitamin B6^a^, Mean ± SD	1.86 ± 1.57	1.89 ± 1.67	1.74 ± 1.13	0.030
Total folate^a^, Mean ± SD	359.27 ± 219.72	364.50 ± 224.21	339.76 ± 201.08	0.037
Folic acid^a^, Mean ± SD	161.28 ± 156.60	161.52 ± 158.70	160.40 ± 148.66	0.957
Food folate^a^, Mean ± SD	198.16 ± 124.77	203.16 ± 129.80	179.48 ± 101.80	0.003
Folate(DFE)^a^, Mean ± SD	472.04 ± 316.79	477.42 ± 321.93	451.96 ± 296.24	0.088
Total choline^a^, Mean ± SD	323.88 ± 191.64	326.43 ± 195.87	314.39 ± 174.79	0.357
Vitamin B12^a^, Mean ± SD	4.86 ± 8.49	4.95 ± 9.26	4.55 ± 4.58	0.780
Added vitamin B12^a^, Mean ± SD	0.73 ± 2.20	0.72 ± 2.29	0.75 ± 1.83	0.039
Vitamin C^a^, Mean ± SD	74.65 ± 83.18	75.96 ± 85.07	69.74 ± 75.61	0.156
Vitamin K^a^, Mean ± SD	98.85 ± 152.05	102.20 ± 161.70	86.33 ± 107.84	0.105
Calcium^a^, Mean ± SD	836.60 ± 529.90	843.63 ± 528.26	810.39 ± 535.66	0.160
Phosphorus^a^, Mean ± SD	1,244.26 ± 625.51	1,261.34 ± 632.24	1,180.50 ± 595.94	0.005
Magnesium^a^, Mean ± SD	271.52 ± 135.99	275.97 ± 137.87	254.89 ± 127.48	0.001
Iron^a^, Mean ± SD	13.81 ± 8.41	13.79 ± 8.28	13.90 ± 8.87	0.952
Zinc^a^, Mean ± SD	10.99 ± 14.10	10.98 ± 14.08	11.03 ± 14.22	0.367
Copper^a^, Mean ± SD	1.20 ± 1.43	1.22 ± 1.54	1.13 ± 0.90	0.019
Sodium^a^, Mean ± SD	3,277.70 ± 1,763.71	3,307.91 ± 1,794.91	3,164.90 ± 1,638.56	0.130
Potassium^a^, Mean ± SD	2,508.32 ± 1,213.01	2,539.28 ± 1,230.03	2,392.68 ± 1,140.89	0.032
Selenium^a^, Mean ± SD	105.70 ± 63.20	106.97 ± 64.98	100.98 ± 55.86	0.042
Caffeine^a^, Mean ± SD	170.96 ± 224.48	166.99 ± 216.42	185.78 ± 251.98	0.101
Theobromine^a^, Mean ± SD	33.82 ± 75.45	34.76 ± 79.88	30.33 ± 55.81	0.667
Alcohol^a^, Mean ± SD	8.25 ± 24.60	8.94 ± 25.57	5.67 ± 20.38	0.005
Moisture^a^, Mean ± SD	2,722.67 ± 1,409.67	2,745.09 ± 1,430.20	2,638.92 ± 1,328.08	0.213
Vitamin D^a^, Mean ± SD	4.27 ± 5.54	4.31 ± 5.76	4.15 ± 4.63	0.648

SD, standard deviation.

a Student *t*-test.

b Chi-square test.

Compared with participants without CHD, those with CHD had significantly lower intakes of several dietary components, including energy (1,831.58 vs. 2,008.67 kcal, *p* < 0.001), protein (71.01 vs. 76.52 g, *p* = 0.002), carbohydrates (217.45 vs. 240.12 g, p < 0.001), total sugar (94.39 vs. 105.44 g, p < 0.001), dietary fiber (14.57 vs. 15.71 g, *p* = 0.022), total fat (73.43 vs. 78.21 g, *p* = 0.033), polyunsaturated fatty acids (16.38 vs. 17.68 g, *p* = 0.006), alpha-carotene (319.46 vs. 352.58 μg, *p* = 0.039), niacin (21.94 vs. 23.56 mg, p = 0.022), vitamin B6 (1.74 vs. 1.89 mg, *p* = 0.030), total folate (364.50 vs. 339.76 μg, *p* = 0.037), food folate (179.48 vs. 203.16 μg, *p* = 0.003), phosphorus (1,180.50 vs. 1,261.34 mg, *p* = 0.005), magnesium (254.89 vs. 275.97 mg, *p* = 0.001), copper (1.13 vs. 1.22 mg, *p* = 0.019), potassium (2,392.68 vs. 2,539.28 mg, *p* = 0.032), selenium (100.98 vs. 106.97 μg, *p* = 0.042), and alcohol (5.67 vs. 8.94 g, p = 0.005).

In contrast, individuals with CHD had higher intakes of alpha-tocopherol (0.72 vs. 0.48 mg, *p* < 0.033) and added vitamin B12 (0.75 vs. 0.72 μg, *p* = 0.039).

### Association between dietary nutrient mixtures and CHD risk

**Figure 2:**
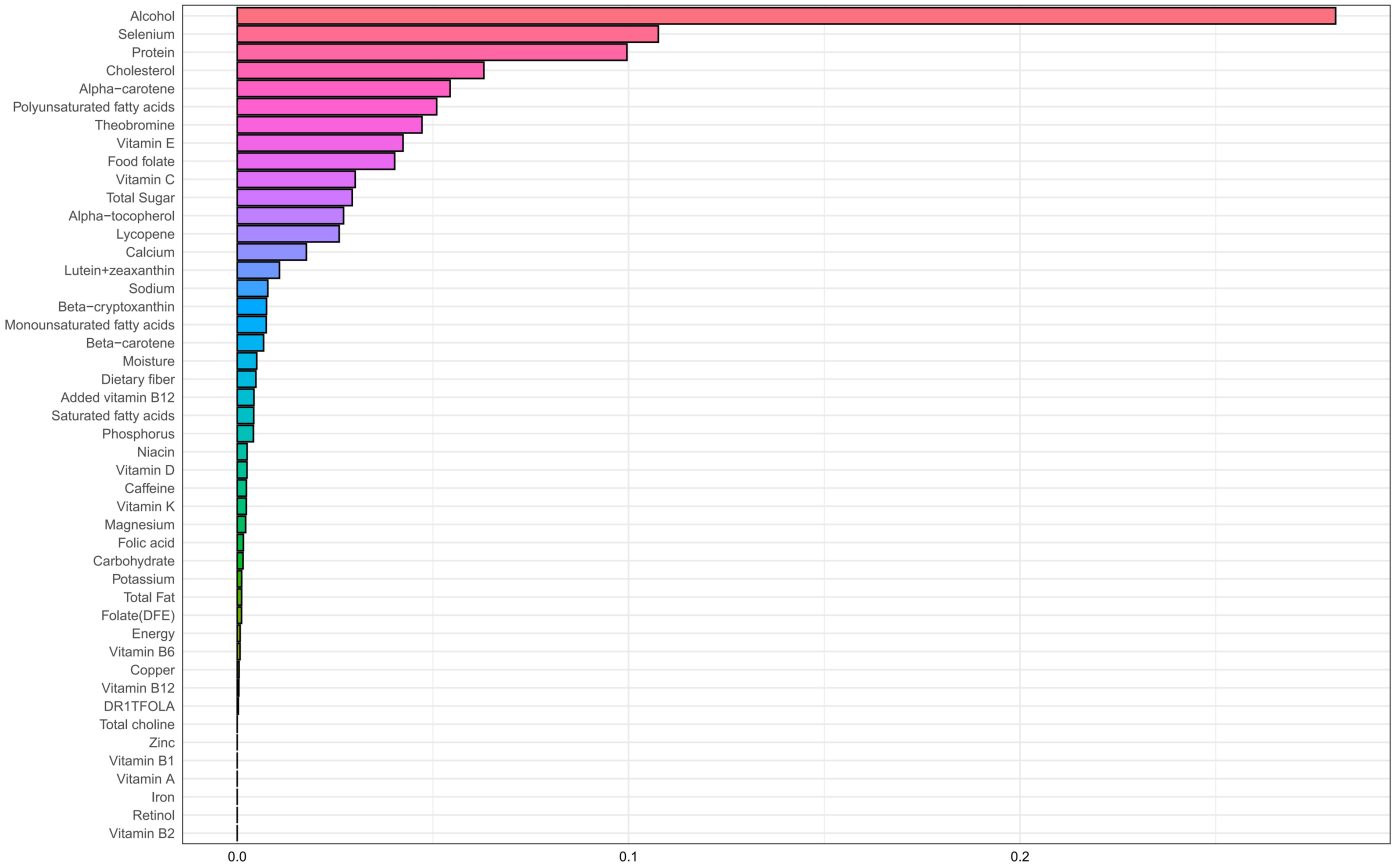
WQS regression weight plot with unconstrained coefficients.

**Figure 3:**
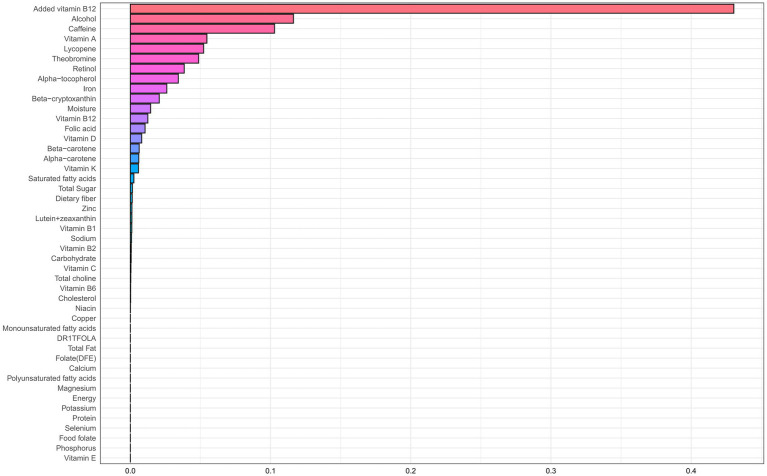
WQS regression weight plot with coefficients constrained to be positive.

Table 2 presents results from the WQS regression, which assessed associations between dietary nutrient mixtures and CHD risk in older adults with accelerated aging. After adjustment for potential confounders (age, sex, race/ethnicity, education, income-to-poverty ratio, BMI, smoking, and alcohol use), an inverse association was observed in the unconstrained model (adjusted OR = 0.90; 95% CI: 0.81–0.99; *p* = 0.048). The primary contributors were alcohol (weight = 0.281), selenium (0.108), protein (0.100), and cholesterol (0.063) (Figure 2; Supplementary Table 1). A similar trend was observed in the positively constrained model (adjusted OR = 0.84; 95% CI: 0.70–1.01; *p* = 0.064), although not statistically significant. Major contributors included added vitamin B12 (0.430), alcohol (0.116), caffeine (0.103), and vitamin A (0.055) (Figure 3; Supplementary Table 2).

**Table 2:** Association between dietary nutrient mixtures and coronary heart disease risk in individuals aged ≥50 years with accelerated aging.

Variable	Estimate	Standard error	z value	OR (95% CI)	P value
WQS-Negative	−0.1066	0.0539	−1.9784	0.90 (0.81, 0.99)	0.048 *
WQS-Positive	−0.1746	0.0943	−1.8518	0.84 (0.70, 1.01)	0.064

### Feature selection for machine learning models

**Figure 4:**
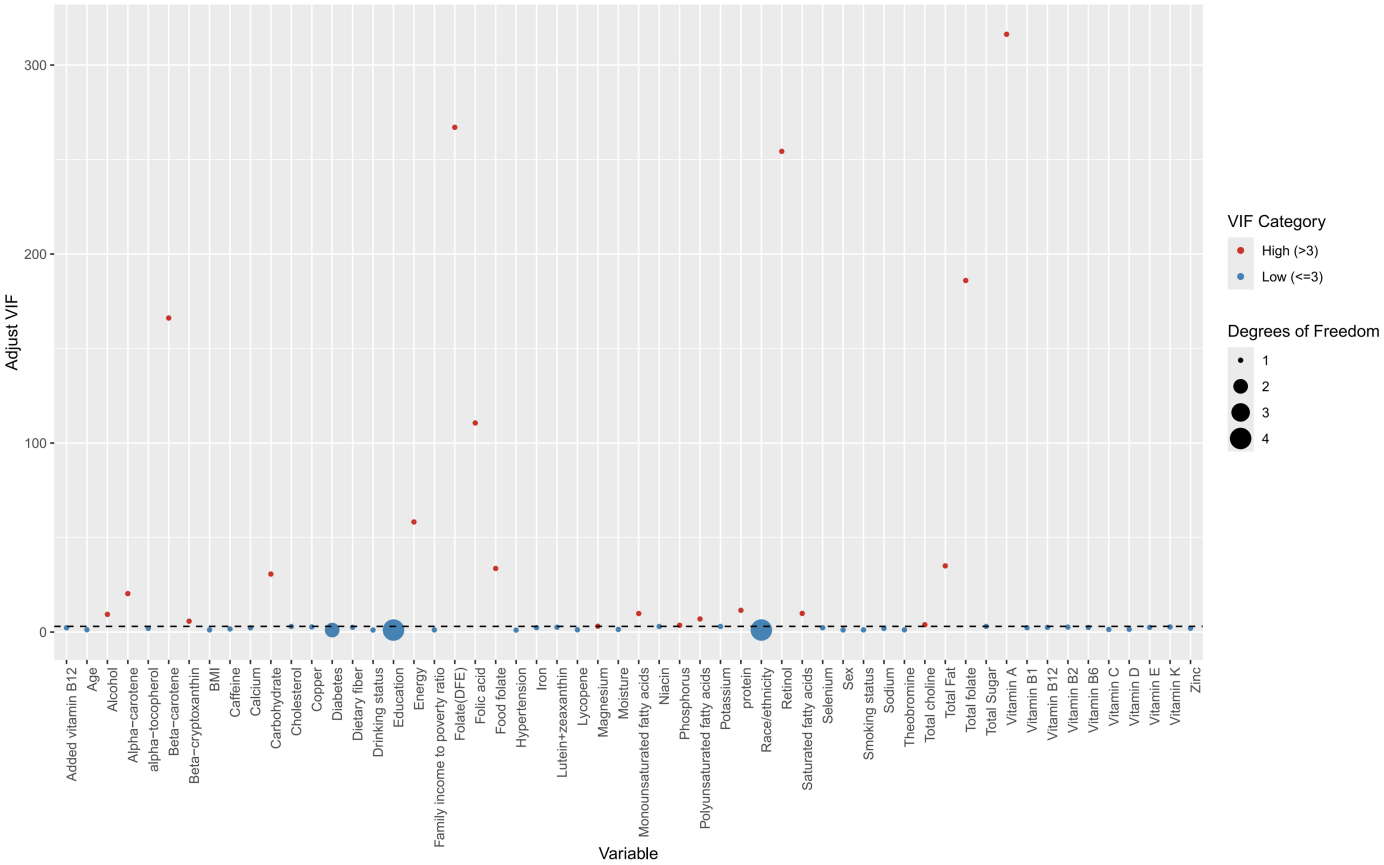
Scatter plot of variance inflation factors (VIFs) across different features. Red points indicate the presence of multicollinearity, while blue points indicate its absence.

Figure 4 displays VIFs used to detect multicollinearity. Variables with adjusted VIFs exceeding 3 were excluded, resulting in the removal of 20 dietary components. These included alcohol, alpha-carotene, beta-carotene, beta-cryptoxanthin, carbohydrates, energy, folate (DFE), folic acid, food folate, magnesium, monounsaturated fatty acids, phosphorus, polyunsaturated fatty acids, protein, retinol, saturated fatty acids, total choline, total fat, total folate, and vitamin A.

**Figure 5:**
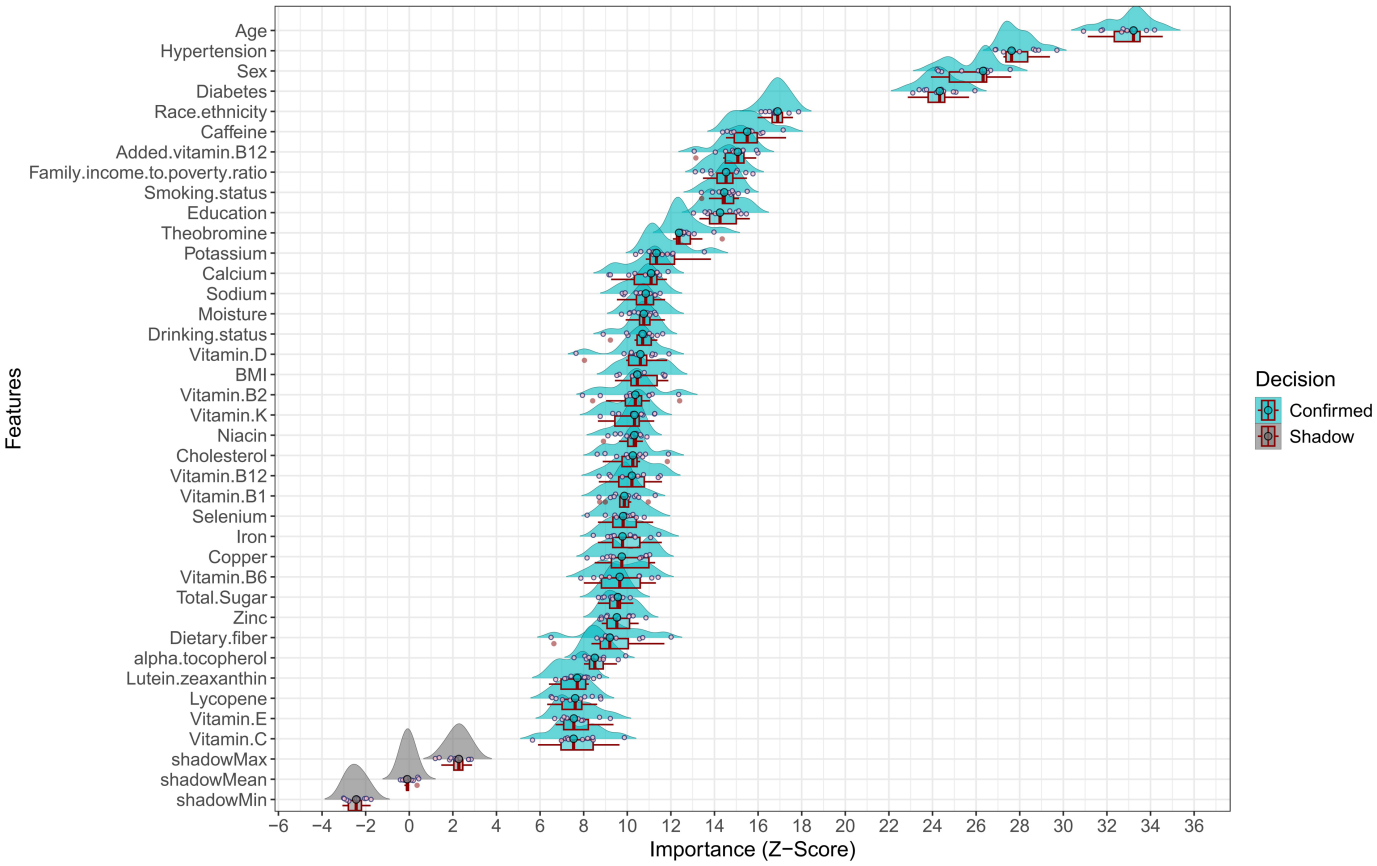
Feature selection results using the BORUTA algorithm.

The BORUTA algorithm then identified 36 variables with significant contributions to the comorbidity of diabetes and hypertension. These included 10 demographic variables (age, hypertension, sex, diabetes, race/ethnicity, income-to-poverty ratio, smoking, education, alcohol use, BMI) and 26 dietary factors (caffeine, added vitamin B12, theobromine, potassium, calcium, sodium, moisture, vitamins D, B2, K, niacin, cholesterol, B12, B1, selenium, iron, copper, B6, total sugar, zinc, dietary fiber, alpha-tocopherol, lutein + zeaxanthin, lycopene, vitamin E, and vitamin C) (Figure 5). Supplementary Figure 2 shows Z-score trends across iterations for feature selection.

### Construction and evaluation of machine learning models

Figures 6 and 7 present heatmaps for six machine learning models: Random Forest, LightGBM, K-KNN, Naive Bayes, SVM, and XGBoost. These models were trained and validated using demographic and dietary variables. Performance was evaluated using AUC-ROC (Figures 8–10), AUC-PR (Figures 11–13), accuracy (Figure 14), F beta score (Figure 15), sensitivity (Supplementary Figure 1), and specificity (Supplementary Figure 2).

**Figure 6:**
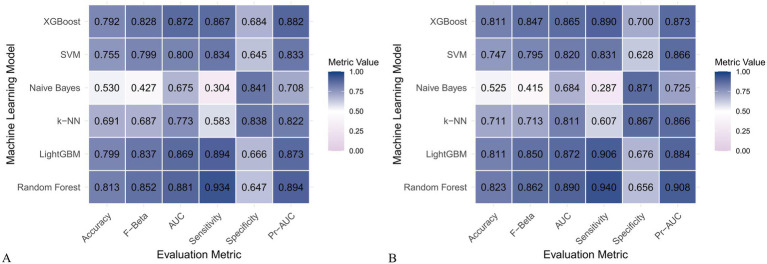
Heatmap comparing the performance of six machine learning models incorporating both demographic characteristics and dietary nutrients. **(A)** Training set; **(B)** Validation set.

**Figure 7:**
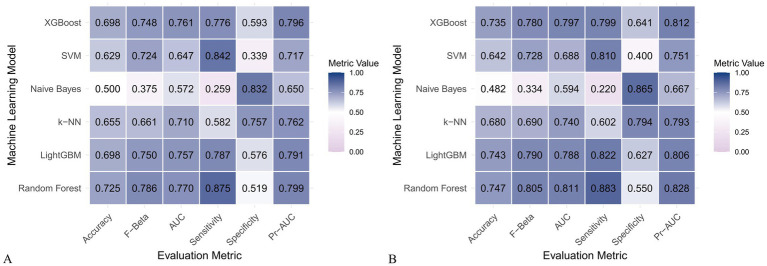
Heatmap comparing the performance of six machine learning models using only dietary nutrients. **(A)** Training set; **(B)** Validation set.

**Figure 8:**
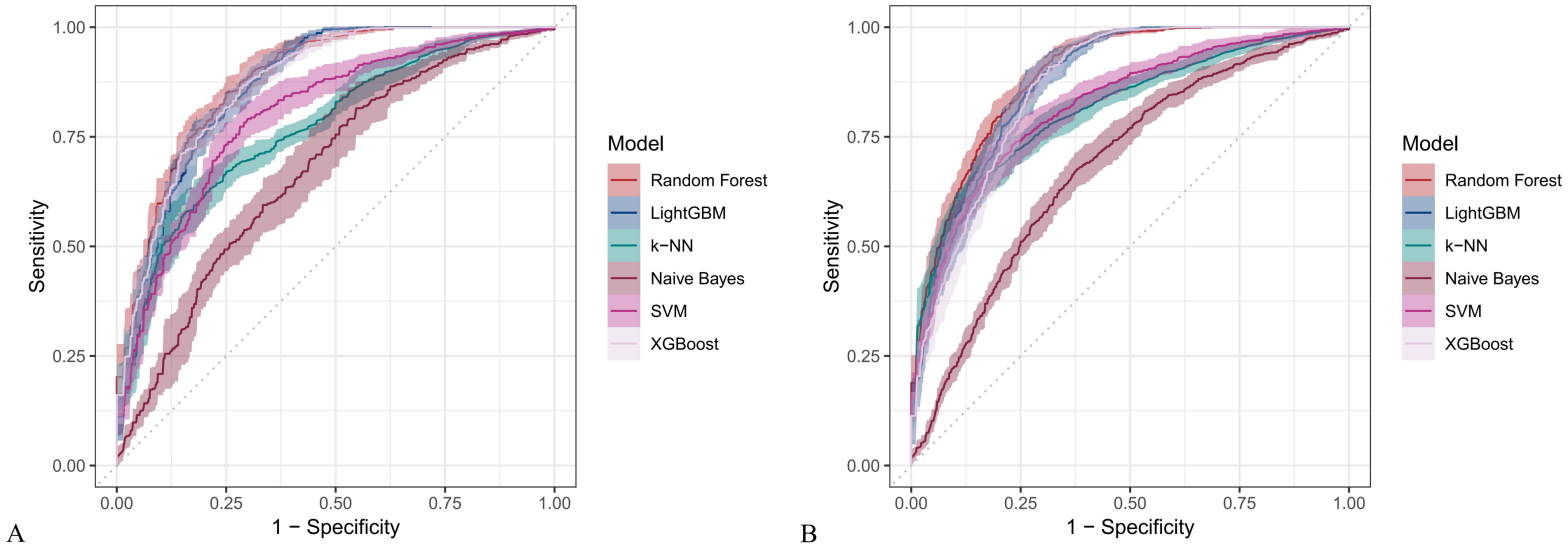
Receiver operating characteristic (ROC) curves for six machine learning models incorporating both demographic characteristics and dietary nutrients. **(A)** Training set; **(B)** Validation set.

**Figure 9:**
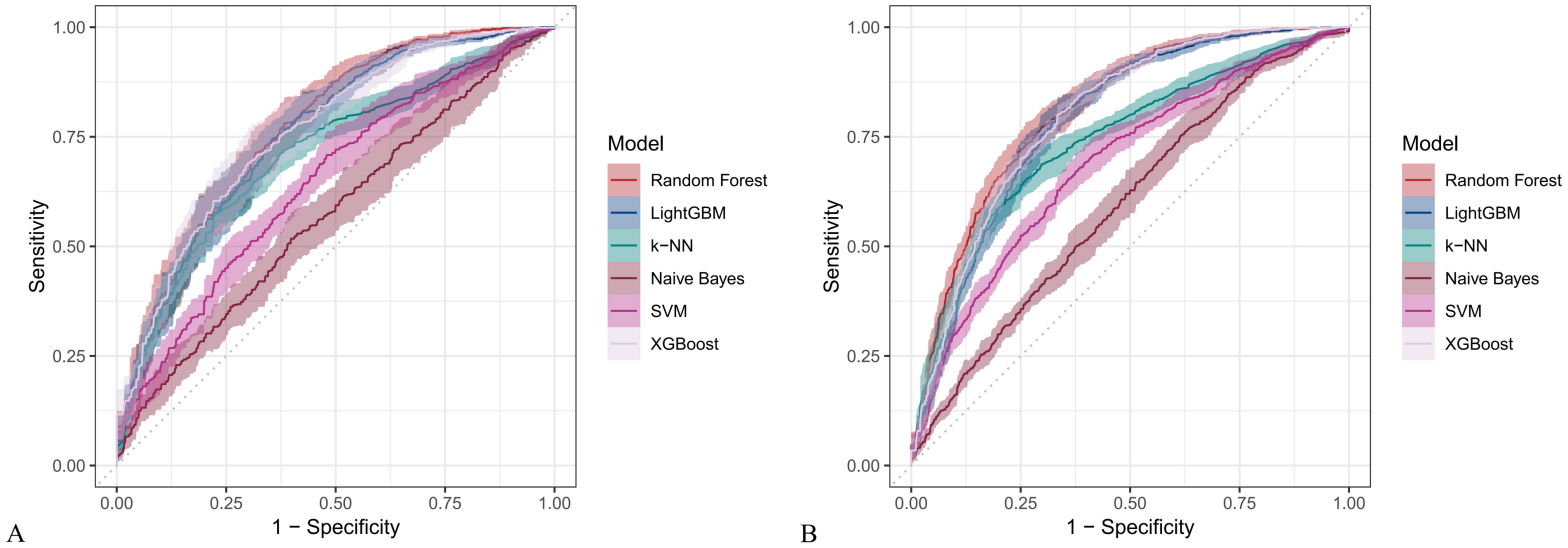
ROC curves for six machine learning models using only dietary nutrients. **(A)** Training set; **(B)** Validation set.

**Figure 10:**
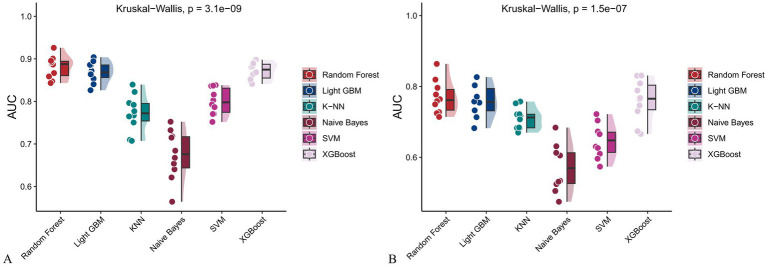
Raincloud plots showing the area under the ROC curve (AUC) for six machine learning models. **(A)** With both demographic characteristics and dietary nutrients; **(B)** With only dietary nutrients.

**Figure 11:**
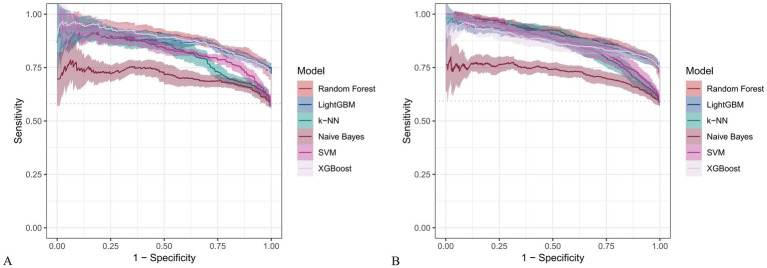
Precision-recall (PR) curves for six machine learning models incorporating both demographic characteristics and dietary nutrients. **(A)** Training set; **(B)** Validation set.

**Figure 12:**
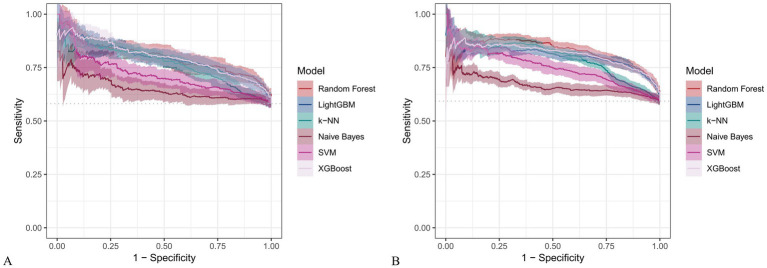
PR curves for six machine learning models using only dietary nutrients. **(A)** Training set; **(B)** Validation set.

**Figure 13:**
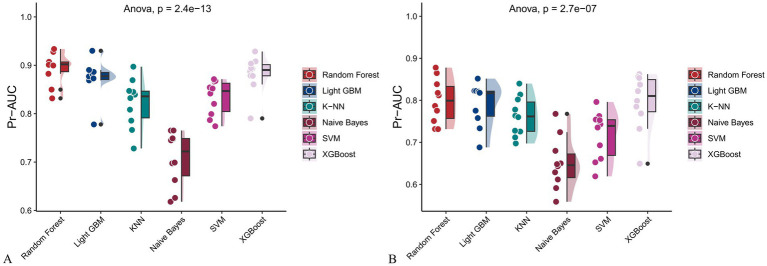
Raincloud plots of PR-AUC scores for six machine learning models. **(A)** Incorporating both demographic characteristics and dietary nutrients; **(B)** Using only dietary nutrients.

**Figure 14:**
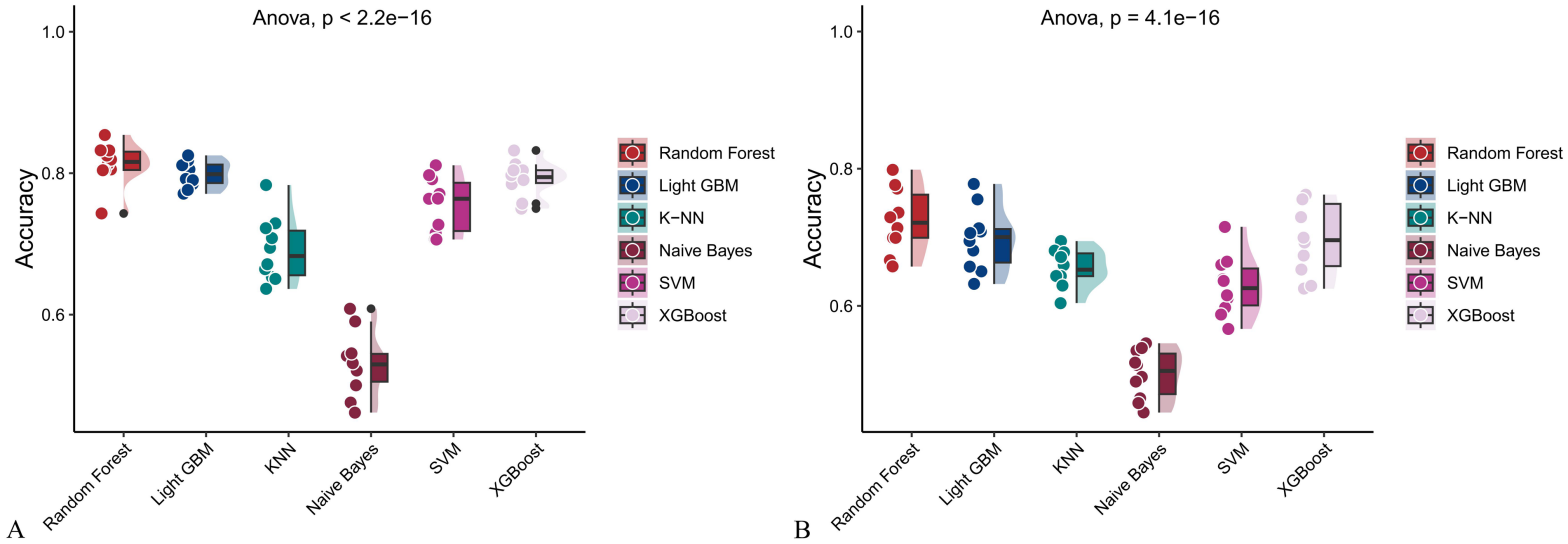
Raincloud plots displaying model accuracy for six machine learning algorithms. **(A)** Models incorporating both demographic characteristics and dietary nutrients; **(B)** Models using only dietary nutrients.

**Figure 15:**
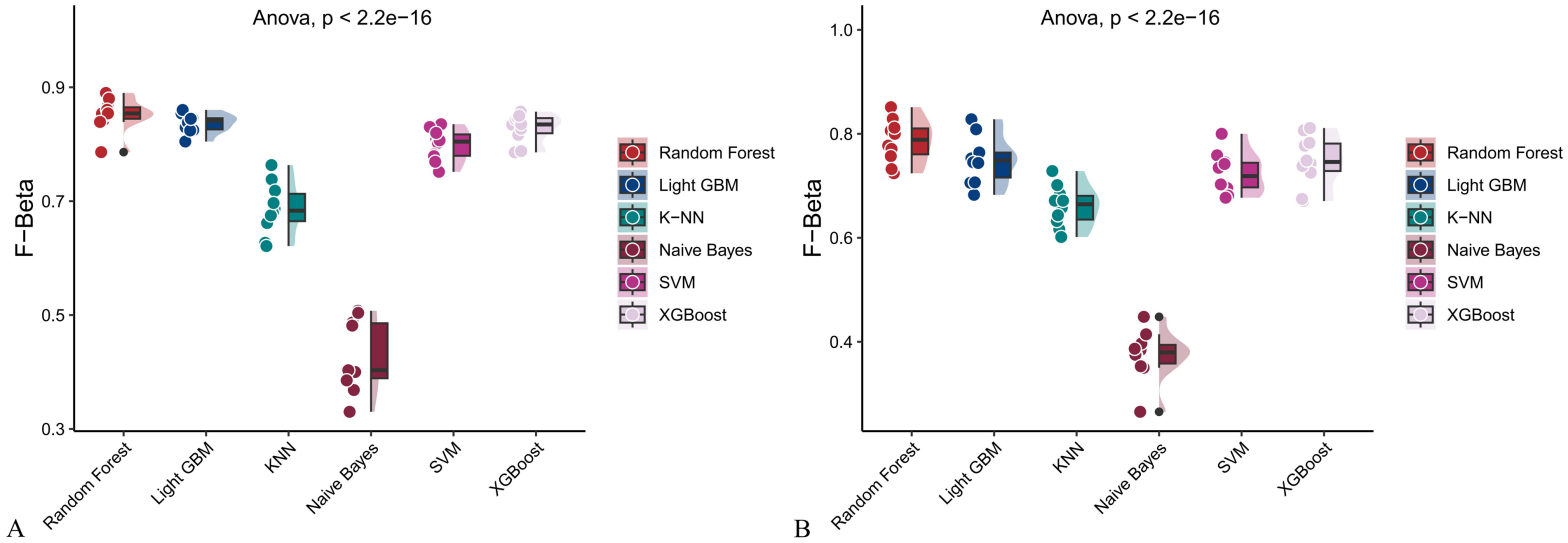
Raincloud plots of F-beta scores across six machine learning models. **(A)** Incorporating both demographic characteristics and dietary nutrients; **(B)** Using only dietary nutrients.

In the training set, Random Forest achieved the best performance across all metrics: accuracy (0.813), F beta (0.852), AUC-ROC (0.881), sensitivity (0.934), specificity (0.647), and AUC-PR (0.894). XGBoost and LightGBM followed closely, with AUC-ROC values of 0.872 and 0.869, AUC-PR of 0.882 and 0.873, accuracy of 0.792 and 0.799, and F-beta scores of 0.828 and 0.837. K-KNN and SVM showed moderate performance, while Naive Bayes had the lowest metrics, particularly AUC-ROC (0.675) and AUC-PR (0.708) (Table 3). In the validation set, Random Forest again outperformed other models, with accuracy (0.823), F beta (0.862), AUC-ROC (0.890), sensitivity (0.940), specificity (0.656), and AUC-PR (0.908) (Table 4), confirming its superior generalizability.

**Table 3:** Performance metrics of six machine learning models in the training set incorporating both demographic characteristics and dietary nutrients.

Model	Accuracy	F Beta	Area under the ROC curve	Sensitivity	Specificity	Area under the PR curve
Random Forest	0.813	0.852	0.881	0.934	0.647	0.894
Light GBM	0.799	0.837	0.869	0.894	0.666	0.873
K-KNN	0.691	0.687	0.773	0.583	0.838	0.822
Naive Bayes	0.530	0.427	0.675	0.304	0.841	0.708
SVM	0.755	0.799	0.800	0.834	0.645	0.833
XGBoost	0.792	0.828	0.872	0.867	0.684	0.882
*p*	<.001^a^	<.001^a^	<.001^b^	<.001^a^	<.001^a^	<.001^a^

a ANOVA test.

b Kruskal-Wallis.

**Table 4:** Performance metrics of six machine learning models in the validation set incorporating both demographic characteristics and dietary nutrients.

Model	Accuracy	F Beta	Area under the ROC curve	Sensitivity	Specificity	Area under the PR curve
Random Forest	0.823	0.862	0.890	0.940	0.656	0.908
Light GBM	0.811	0.850	0.872	0.906	0.676	0.884
K-KNN	0.711	0.713	0.811	0.607	0.867	0.866
Naive Bayes	0.525	0.415	0.684	0.287	0.871	0.725
SVM	0.747	0.795	0.820	0.831	0.628	0.866
XGBoost	0.811	0.847	0.865	0.890	0.700	0.873
*p*	<.001^a^	<.001^a^	<.001^b^	<.001^a^	<.001^a^	<.001^a^

a ANOVA test.

b Kruskal-Wallis.

When only dietary variables were used, Random Forest remained the best-performing model. In the training set, it achieved an accuracy of 0.725, F beta of 0.786, AUC-ROC of 0.770, sensitivity of 0.875, specificity of 0.519, and AUC-PR of 0.799. XGBoost and LightGBM showed comparable results: AUC-ROC of 0.761 and 0.757, AUC-PR of 0.796 and 0.791, accuracy of 0.698, and F beta scores of 0.748 and 0.750 (Table 5). In the validation set, Random Forest again led all metrics: accuracy (0.747), F beta (0.805), AUC-ROC (0.811), sensitivity (0.883), specificity (0.550), and AUC-PR (0.828) (Table 6).

**Table 5:** Performance metrics of six machine learning models in the training set using only dietary nutrients.

Model	Accuracy	F Beta	Area under the ROC curve	Sensitivity	Specificity	Area under the PR curve
Random Forest	0.725	0.786	0.770	0.875	0.519	0.799
Light GBM	0.698	0.750	0.757	0.787	0.576	0.791
K-KNN	0.655	0.661	0.710	0.582	0.757	0.762
Naive Bayes	0.500	0.375	0.572	0.259	0.832	0.650
SVM	0.629	0.724	0.647	0.842	0.339	0.717
XGBoost	0.698	0.748	0.761	0.776	0.593	0.796
*p*	<.001^a^	<.001^a^	<.001^b^	<.001^a^	<.001^a^	<.001^a^

a ANOVA test.

b Kruskal-Wallis.

**Table 6:** Performance metrics of six machine learning models in the validation set using only dietary nutrients.

Model	Accuracy	F Beta	Area under the ROC curve	Sensitivity	Specificity	Area under the PR curve
Random Forest	0.747	0.805	0.811	0.883	0.550	0.828
Light GBM	0.743	0.790	0.788	0.822	0.627	0.806
K-KNN	0.680	0.690	0.740	0.602	0.794	0.793
Naive Bayes	0.482	0.334	0.594	0.220	0.865	0.667
SVM	0.642	0.728	0.688	0.810	0.400	0.751
XGBoost	0.735	0.780	0.797	0.799	0.641	0.812
*p*	<.001^a^	<.001^a^	<.001^b^	<.001^a^	<.001^a^	<.001^a^

a ANOVA test.

b Kruskal-Wallis.

Across all analyses, Random Forest consistently demonstrated superior performance regardless of input variable type. Statistically significant differences in model performance were observed in all comparisons (*p* < 0.001) (Tables 3–6).

### Interpretation of feature importance using SHAP and LIME

The SHAP algorithm was employed to interpret feature contributions to CHD risk prediction in individuals over age 50 with accelerated aging. Two scenarios were considered: one including both demographic and dietary variables, and one using dietary variables alone. Supplementary Figure 3 illustrates the top 25 features under each scenario using the Random Forest model, with SHAP values quantifying each feature’s importance.

When both demographic and dietary variables were included, Age (SHAP = 0.0509) and Hypertension (0.0472) were the strongest positive contributors. Negative contributors included vitamin B12 (0.0107), lycopene (0.0101), potassium (0.0097), total sugar (0.0081), and lutein + zeaxanthin (0.0075). In the dietary-only model, vitamin B12 (0.0365), lycopene (0.0256), theobromine (0.0193), total sugar (0.0187), and lutein + zeaxanthin (0.0160) had the strongest negative contributions, while caffeine (0.0231) and cholesterol (0.0113) contributed positively.

Force plots and waterfall plots (Supplementary Figures 4, 5) were used to visualize individual-level predictions. In the combined model, the baseline CHD prediction was 0.584, increasing to 0.889 after accounting for feature contributions. In the dietary-only model, the baseline was 0.593 and rose to 0.737 after feature inclusion.

SHAP interaction dependency plots further illustrated the nonlinear relationships between key dietary nutrients and CHD risk (Supplementary Figures 6, 7). In the fully adjusted model, higher intakes of caffeine, lycopene, potassium, total sugar, vitamin B12, and lutein + zeaxanthin were associated with lower SHAP values, indicating protective effects, with notable interactions by age, sex, and hypertension status (Supplementary Figure 6). Similar trends were observed in the unadjusted model using dietary variables alone, though effect magnitudes were slightly attenuated (Supplementary Figure 7). LIME explanations for individual predictions (Supplementary Figures 8, 9) consistently identified vitamin B6, dietary fiber, zinc, vitamin B12, and lutein + zeaxanthin as key negative contributors to CHD risk, reinforcing the robustness of these findings across adjusted and unadjusted models.

## Discussion

Using data from NHANES and multiple machine learning models, this study explored the relationship between dietary nutrient intake and the risk of CHD in adults aged 50 and above who exhibit signs of accelerated aging. After adjusting for potential confounders, we found that specific combinations of dietary nutrients were associated with a reduced risk of CHD. Among the models evaluated, the random forest model demonstrated superior predictive performance. Interpretation through SHAP and LIME revealed that higher intakes of vitamin B12 and lutein + zeaxanthin were inversely associated with CHD risk. These findings suggest that both nutrients may offer protective benefits against CHD in older adults experiencing accelerated aging.

The inverse association between vitamin B12 and CHD risk aligns with substantial evidence on its role in homocysteine metabolism. Vitamin B12 deficiency can elevate homocysteine levels—a known cardiovascular risk factor due to its effects on endothelial dysfunction and atherosclerosis (24, 25). Huang et al. reported that vitamin B12 deficiency, commonly observed in older adults due to reduced absorption, increases cardiovascular risk, particularly in individuals with dietary restrictions such as vegetarians (26). A large-scale population-based study further supported these findings by showing that adequate vitamin B12 intake may help reduce cardiovascular events (27). These results highlight the importance of maintaining sufficient vitamin B12 intake for cardiovascular health, especially among aging individuals.

Similarly, lutein and zeaxanthin—carotenoids with strong antioxidant properties—have been associated with cardiovascular protection. Nicolantonio et al. found that these compounds may reduce CHD risk by attenuating oxidative stress and inflammation (28). A Swedish study also found significantly lower plasma levels of lutein and zeaxanthin in patients with coronary artery disease compared to healthy controls, further reinforcing their protective potential (29). Our findings are consistent with these observations, showing that higher dietary intake of these carotenoids is linked to reduced CHD risk. Their mechanisms—such as neutralizing free radicals and lowering inflammatory markers like interleukin-6—are especially relevant in the context of accelerated aging (30).

However, some discrepancies remain. For example, a prospective cohort study by Zhang et al. using NHANES data found no significant association between serum vitamin B12 levels and mortality in patients with existing CHD (31). Interestingly, the study identified methylmalonic acid (MMA), a marker of functional vitamin B12 deficiency, as a stronger predictor of cardiovascular mortality. This suggests that functional status may be more relevant than serum levels. Our study assessed dietary intake rather than serum concentrations, which may more accurately reflect long-term adequacy and could explain the differing results. Furthermore, the application of machine learning in our study allowed us to capture complex, non-linear associations that might be missed by traditional statistical methods.

With regard to lutein and zeaxanthin, findings from the Age-Related Eye Disease Study 2 (AREDS2) indicated that supplementation with these carotenoids did not significantly reduce cardiovascular events in older adults with age-related macular degeneration (32). This contrasts with our results, which highlight a protective association with dietary intake. The discrepancy may stem from differences in study populations; participants in AREDS2 had a specific ocular disease and may not represent older adults experiencing accelerated aging. Additionally, nutrients consumed in whole foods may exert effects through synergistic interactions not replicated by supplements (33). Differences in bioavailability between dietary and supplemental forms may also contribute to inconsistent outcomes (34).

A particularly notable finding from our analysis is the strong protective role of vitamin B12 and lutein + zeaxanthin in reducing CHD risk among individuals with accelerated aging. While previous research has demonstrated cardiovascular benefits of these nutrients, their specific impact in this high-risk subgroup has been underexplored. Our findings emphasize their potential as practical dietary targets for individuals with advanced biological aging. Focusing on these nutrients may aid in the development of personalized nutritional interventions to reduce CHD risk and improve health outcomes in this vulnerable population.

By linking specific dietary nutrients to reduced CHD risk within the framework of accelerated aging, our findings contribute to the broader field of nutritional epidemiology (5). Accelerated aging is characterized by heightened oxidative stress and inflammation—both central to CHD pathogenesis (35, 36). The COVID-19 pandemic has highlighted the critical interplay between nutrition, inflammation, and cardiovascular health (37, 38). Research also underscores that maintaining optimal levels of key nutrients is critical for mitigating inflammation and oxidative stress to enhance immune function in COVID-19 patients, given that these two processes are shared foundational mechanisms for both chronic diseases, such as coronary heart disease, and susceptibility to severe infections (39). Vitamin B12 lowers homocysteine levels, supporting vascular health, while lutein and zeaxanthin provide antioxidant protection that mitigates oxidative damage, a hallmark of aging and cardiovascular disease (12). These mechanisms reinforce the relevance of our results and support dietary strategies tailored to biological aging. This aligns with the principles of personalized nutrition, which advocates for customizing dietary recommendations based on an individual’s physiological age and health status (40). Our findings offer a straightforward strategy for clinical practice: advising high-risk older adults, identified by phenotypic age acceleration, to consume more foods rich in vitamin B12 (such as fish, meat, and dairy products) and lutein + zeaxanthin (such as spinach, kale, and corn).

Several limitations of this study should be acknowledged. First, the cross-sectional nature of NHANES data limits causal inference, and reverse causality remains possible—individuals with CHD may have changed their dietary habits. Second, although machine learning models such as random forests can detect complex, non-linear patterns, their interpretability is limited, even with tools like SHAP and LIME. Third, residual confounding from unmeasured factors—such as genetics or socioeconomic status—may have influenced the observed associations. Fourth, dietary data were based on two 24-h recalls, which may not accurately reflect habitual intake and are subject to recall bias. Lastly, since our analysis is based on NHANES data, generalizability may be limited, particularly for older adults in different cultural or geographic settings.

## Conclusion

In conclusion, this study shows that higher dietary intakes of vitamin B12 and lutein + zeaxanthin are associated with a lower risk of coronary heart disease in older adults experiencing accelerated aging. These findings provide valuable insights for developing targeted dietary strategies. Future research should aim to confirm these associations through longitudinal cohorts and more diverse populations such as COVID-19 patients, explore the underlying mechanisms, confirm causality and support personalized nutrition strategies and evaluate their generalizability across diverse populations.

## Data Availability

The raw data supporting the conclusions of this article will be made available by the authors, without undue reservation.

## Supplementary material

SUPPLEMENTARY TABLE 1 Weights of dietary nutrient mixtures in WQS regression with unconstrained coefficients.

SUPPLEMENTARY TABLE 2 Weights of dietary nutrient mixtures in WQS regression with coefficients constrained to be positive.

SUPPLEMENTARY FIGURE 1 Raincloud plots of model sensitivity for six machine learning models. **(A)** With both demographic characteristics and dietary nutrients; **(B)** With only dietary nutrients.

SUPPLEMENTARY FIGURE 2 Raincloud plots of model specificity for six machine learning models. **(A)** With both demographic characteristics and dietary nutrients; **(B)** With only dietary nutrients. SUPPLEMENTARY FIGURE 3 SHAP (SHapley Additive exPlanations) analysis of feature importance in the best-performing model. **(A)** Considering both demographic characteristics and dietary nutrients; **(B)** Considering only dietary nutrients.

SUPPLEMENTARY FIGURE 4 Force and waterfall plots showing individual-level predictions and feature contributions for the best-performing model incorporating both demographic characteristics and dietary nutrients.

SUPPLEMENTARY FIGURE 5 Force and waterfall plots showing individual-level predictions and feature contributions for the best-performing model using only dietary nutrients.

SUPPLEMENTARY FIGURE 6 SHAP interaction dependency plots illustrating the relationships between the top six dietary nutrients and SHAP values, considering both demographic characteristics and dietary nutrients.

SUPPLEMENTARY FIGURE 7 SHAP interaction dependency plots illustrating the relationships between the top six dietary nutrients and SHAP values, considering only dietary nutrients.

SUPPLEMENTARY FIGURE 8 Local Interpretable Model-Agnostic Explanations (LIME) for individual predictions from the best-performing model incorporating both demographic characteristics and dietary nutrients.

SUPPLEMENTARY FIGURE 9 LIME analysis for individual predictions from the best-performing model using only dietary nutrients.

SUPPLEMENTARY FIGURE 10 Bar plot illustrating class imbalance before and after Synthetic Minority Over-sampling Technique (SMOTE) processing.

SUPPLEMENTARY FIGURE 11 Trends in standardized Z-scores of selected features throughout the BORUTA selection process
