# Supplementary material for: Interpretable prediction of coronary heart disease risk in adults over 50 with accelerated aging using 45 dietary nutrients
Source: Front Nutr. 2025 Sep 18;12:1666644. doi: 10.3389/fnut.2025.1666644 (PMC12488431; doi:10.3389/fnut.2025.1666644)
Supplement: SUPPLEMENTARY TABLE 1 — Weights of dietary nutrient mixtures in WQS regression with unconstrained coefficients. [file Table_1.DOCX]

| **Mixture Name** | **Mean Weight** |
| --- | --- |
| Alcohol | 0.281 |
| Selenium | 0.108 |
| Protein | 0.100 |
| Cholesterol | 0.063 |
| Alpha-carotene | 0.054 |
| Polyunsaturated fatty acids | 0.051 |
| Theobromine | 0.047 |
| Vitamin E | 0.042 |
| Food folate | 0.040 |
| Vitamin C | 0.030 |
| Total Sugar | 0.029 |
| Alpha-tocopherol | 0.027 |
| Lycopene | 0.026 |
| Calcium | 0.018 |
| Lutein+zeaxanthin | 0.011 |
| Sodium | 0.008 |
| Beta-cryptoxanthin | 0.007 |
| Monounsaturated fatty acids | 0.007 |
| Beta-carotene | 0.007 |
| Moisture | 0.005 |
| Dietary fiber | 0.005 |
| Added vitamin B12 | 0.004 |
| Saturated fatty acids | 0.004 |
| Phosphorus | 0.004 |
| Niacin | 0.003 |
| Vitamin D | 0.003 |
| Caffeine | 0.002 |
| Vitamin K | 0.002 |
| Magnesium | 0.002 |
| Folic acid | 0.002 |
| Carbohydrate | 0.001 |
| Potassium | 0.001 |
| Total Fat | 0.001 |
| Folate(DFE) | 0.001 |
| Energy | 0.001 |
| Vitamin B6 | 0.001 |
| Copper | 0.000 |
| Vitamin B12 | 0.000 |
| DR1TFOLA | 0.000 |
| Total choline | 0.000 |
| Zinc | 0.000 |
| Vitamin B1 | 0.000 |
| Vitamin A | 0.000 |
| Iron | 0.000 |
| Retinol | 0.000 |
| Vitamin B2 | 0.000 |
