# Supplementary figures and images for: Interpretable prediction of coronary heart disease risk in adults over 50 with accelerated aging using 45 dietary nutrients
Source: Front Nutr. 2025 Sep 18;12:1666644. doi: 10.3389/fnut.2025.1666644 (PMC12488431; doi:10.3389/fnut.2025.1666644)

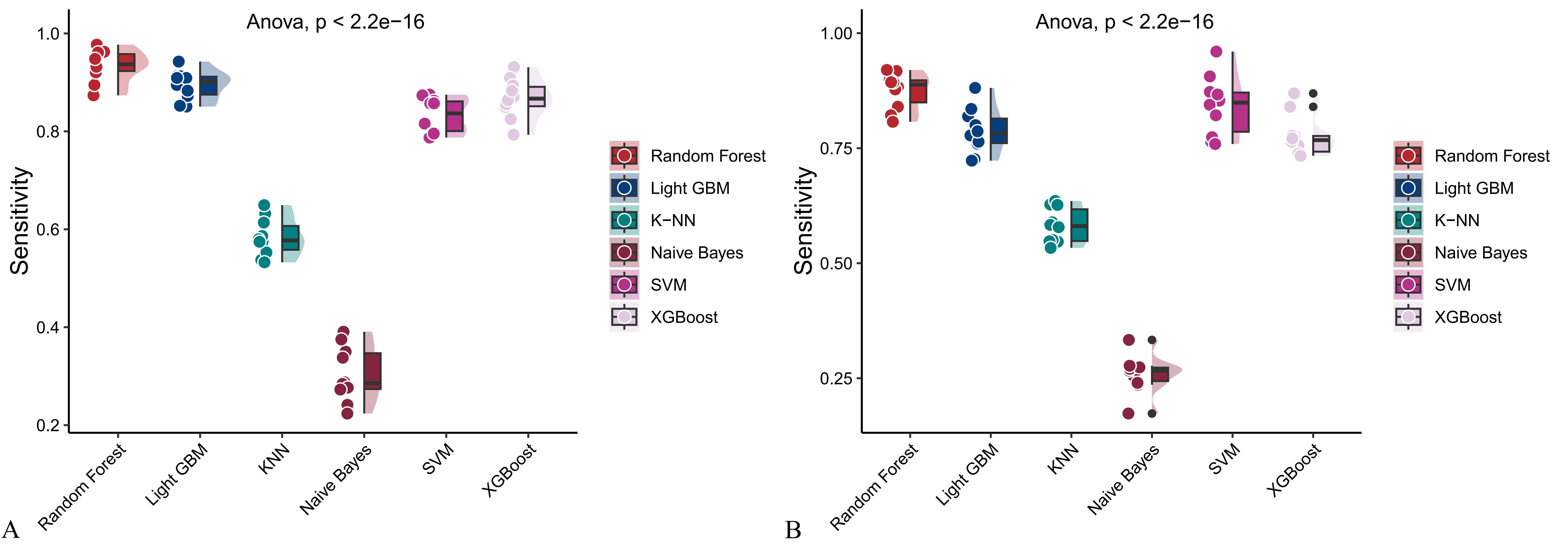

Supplement: SUPPLEMENTARY FIGURE 1 — Raincloud plots of model sensitivity for six machine learning models. (A) With both demographic characteristics and dietary nutrients; (B) With only dietary nutrients. [file Image_1.JPEG]

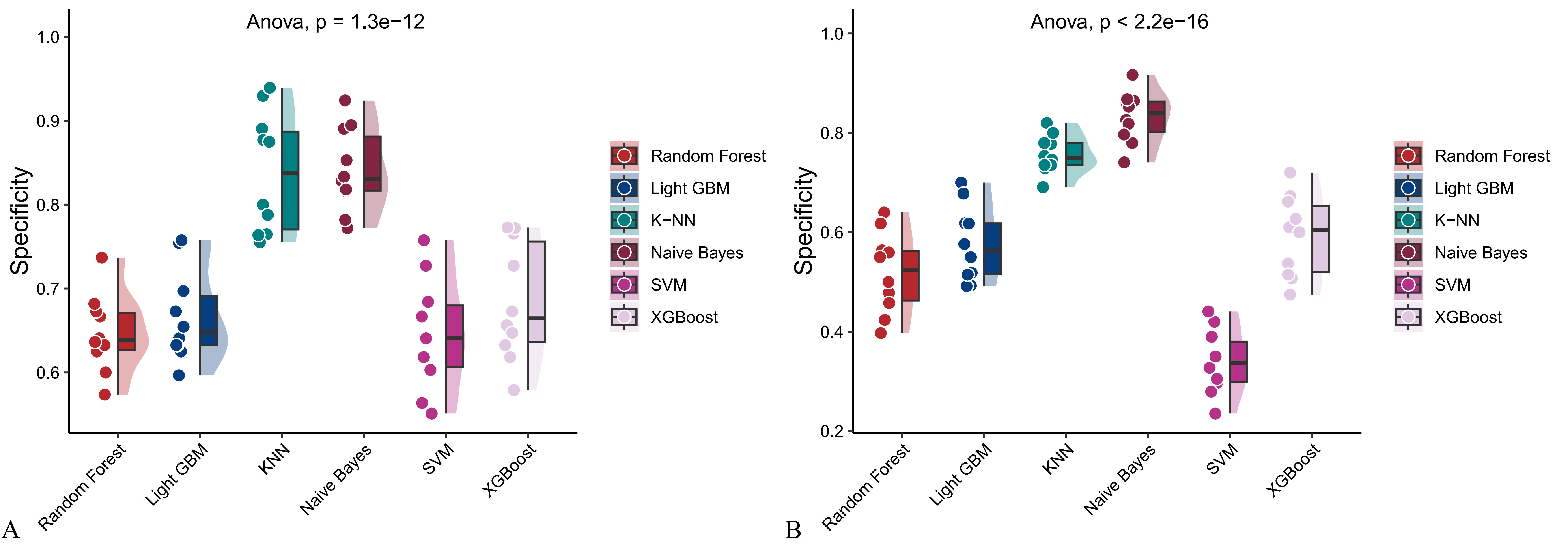

Supplement: SUPPLEMENTARY FIGURE 2 — Raincloud plots of model specificity for six machine learning models. (A) With both demographic characteristics and dietary nutrients; (B) With only dietary nutrients. SUPPLEMENTARY FIGURE 3 SHAP (SHapley Additive exPlanations) analysis of feature importance in the best-performing model. (A) Considering both demographic characteristics and dietary nutrients; (B) Considering only dietary nutrients. [file Image_2.JPEG]

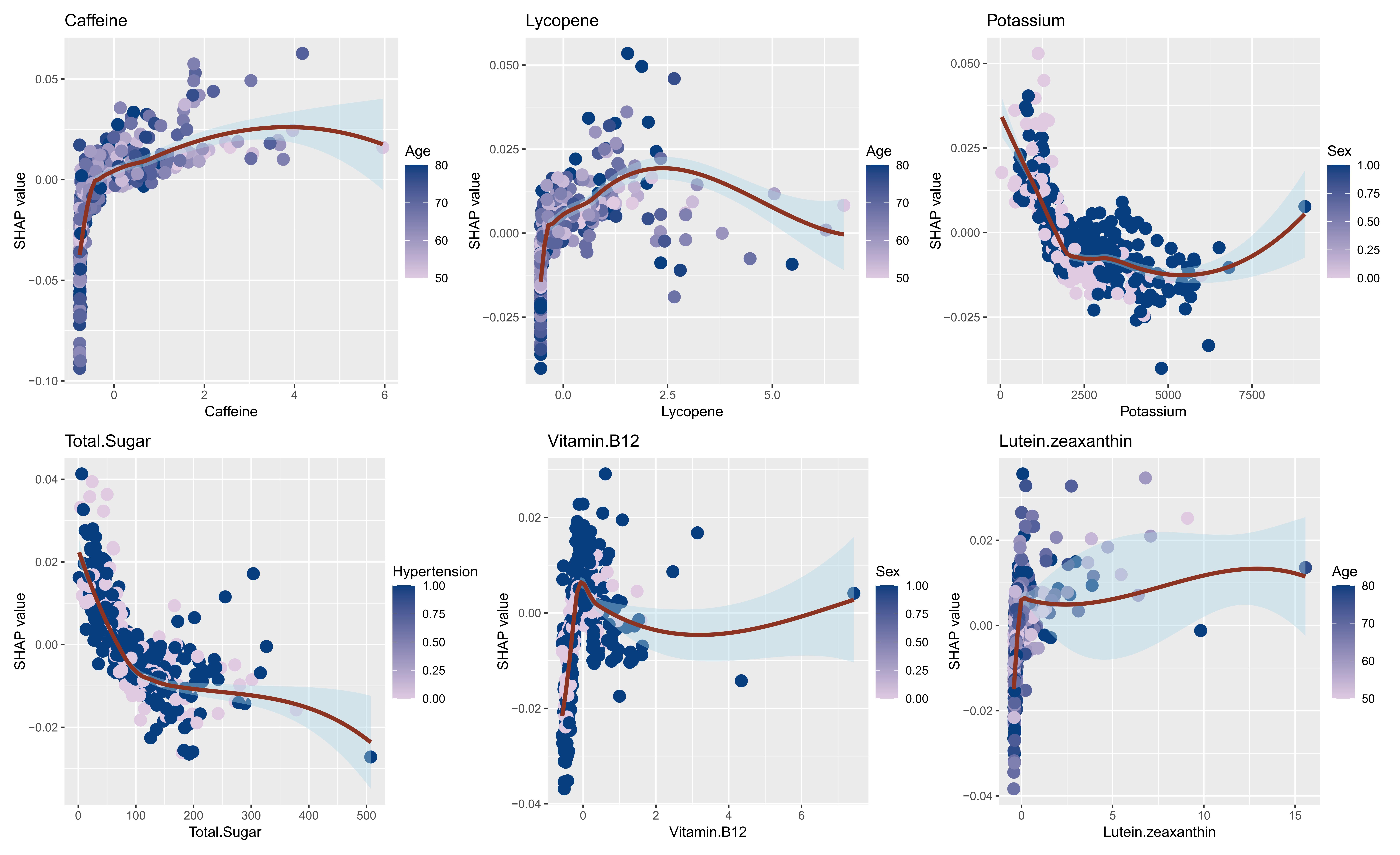

Supplement: SUPPLEMENTARY FIGURE 6 — SHAP interaction dependency plots illustrating the relationships between the top six dietary nutrients and SHAP values, considering both demographic characteristics and dietary nutrients. [file Image_6.JPEG]

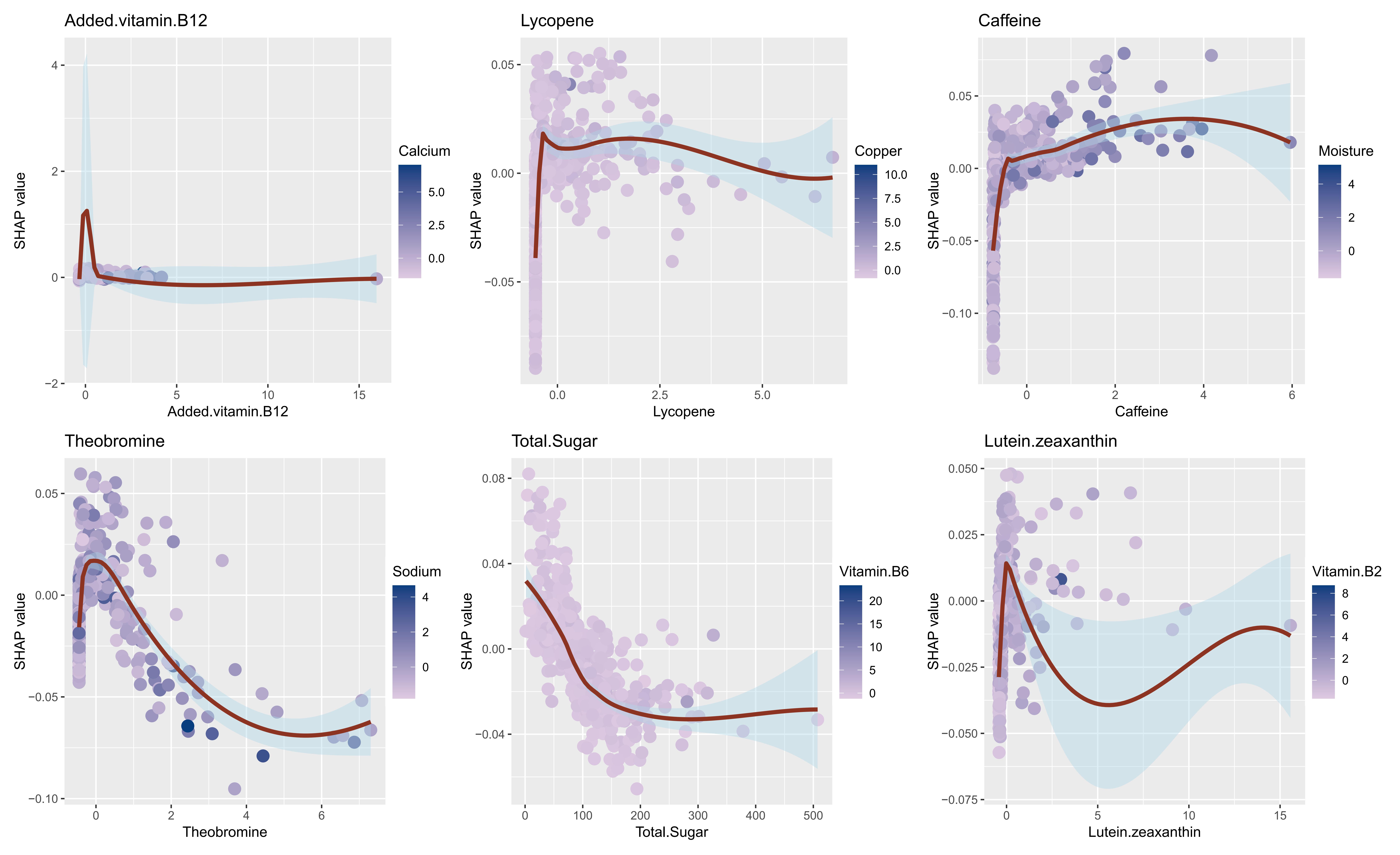

Supplement: SUPPLEMENTARY FIGURE 7 — SHAP interaction dependency plots illustrating the relationships between the top six dietary nutrients and SHAP values, considering only dietary nutrients. [file Image_7.JPEG]

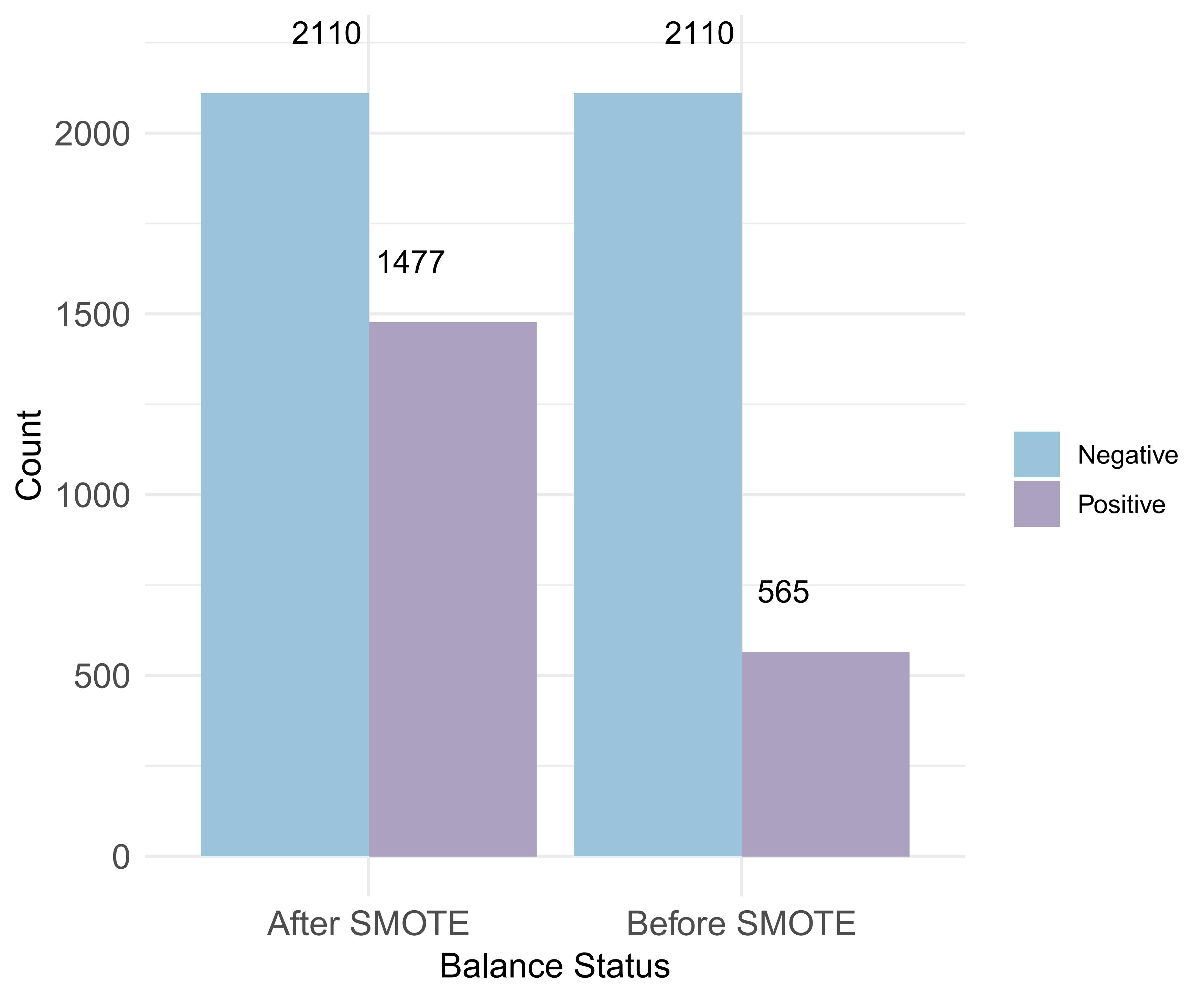

Supplement: SUPPLEMENTARY FIGURE 10 — Bar plot illustrating class imbalance before and after Synthetic Minority Over-sampling Technique (SMOTE) processing. [file Image_10.JPEG]

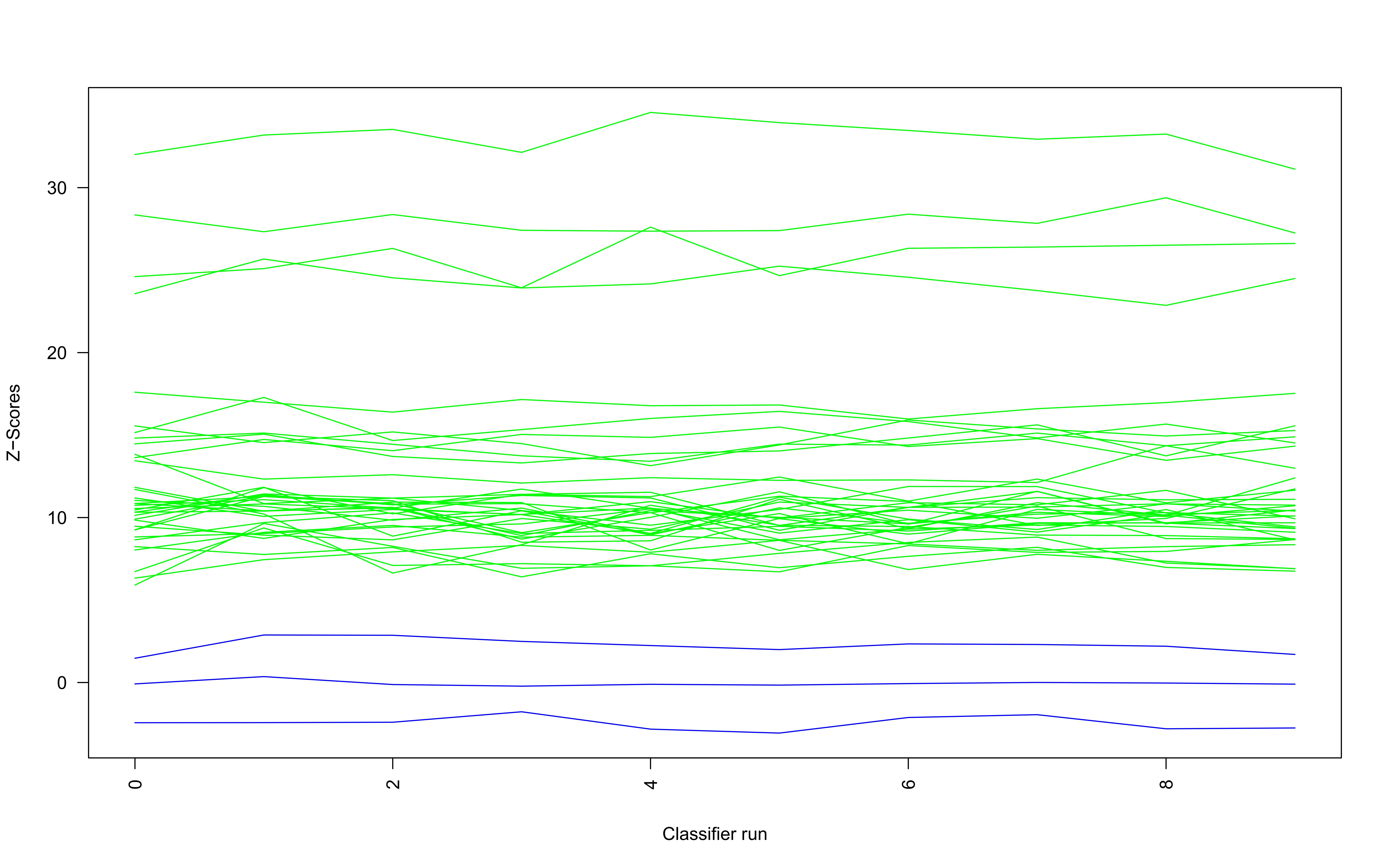

Supplement: SUPPLEMENTARY FIGURE 11 — Trends in standardized Z-scores of selected features throughout the BORUTA selection process [file Image_11.JPEG]

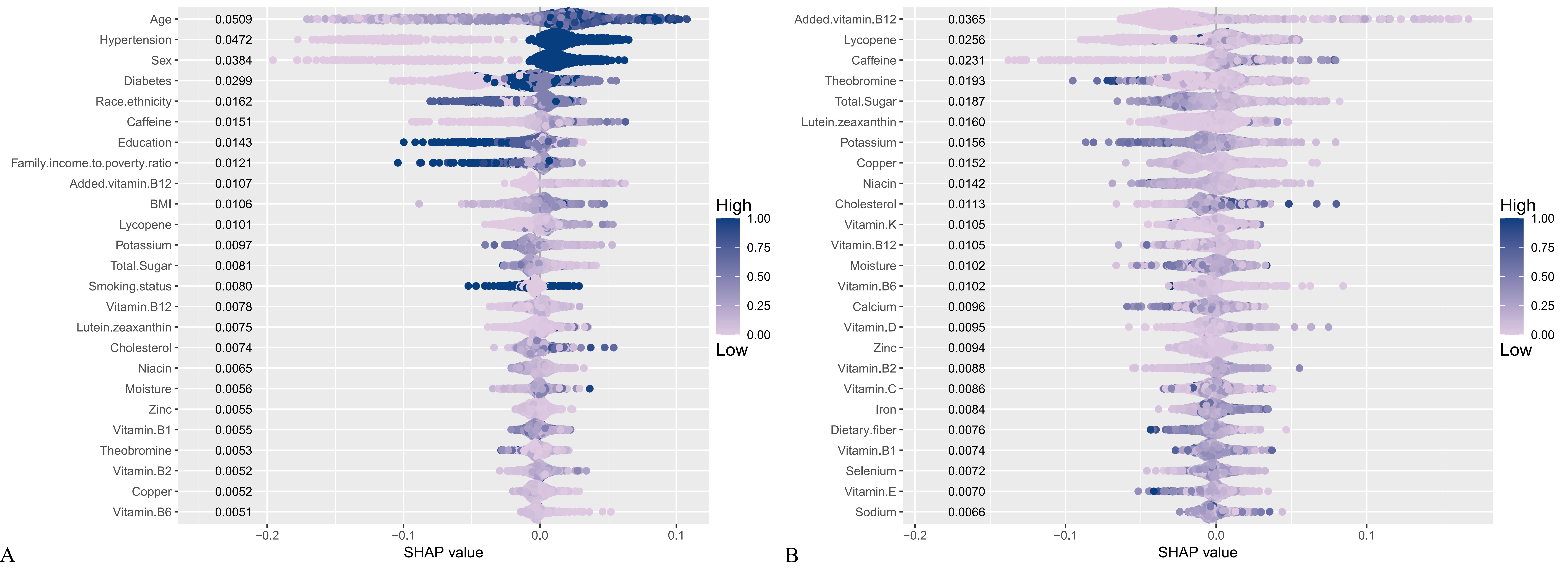

Supplement: Supplementary file 13 [file Image_3.JPEG]
